# Ipsilateral Supernumerary Heads of the Sternocleidomastoid and Flat Fibers of the Omohyoid Muscle: A Cadaveric Case Report

**DOI:** 10.7759/cureus.84931

**Published:** 2025-05-27

**Authors:** Juma Mwalimu Mbwambo

**Affiliations:** 1 Anatomy, Sri Ramachandra Medical College and Research Institute (SRMC&amp;RI), Chennai, IND

**Keywords:** cadaver case report, head and neck surgery, omohyoid, plastic and reconstructive surgery, sternocleidomastoid

## Abstract

This case report presents an ipsilateral anatomical variation in the right neck of an 80-year-old male cadaver. The Sternocleidomastoid muscle exhibited four heads, one sternal and three clavicular heads. All the heads are merging into a common belly and inserting normally into the mastoid process. The right omohyoid muscle appeared as flat fibers, lacking an intermediate tendon and having its superior belly fused with the sternohyoid muscle. Abnormal development of the embryonic branchial arch mesoderm and occipital myotome leads to these variations. The Sternocleidomastoid and omohyoid are critical surgical landmarks in the neck. Their aberrant morphology can distort normal anatomical planes, increasing risks of neurovascular injury during neck dissections, central venous catheterization, or reconstructive surgeries. These variations are distinct and previously unreported in this specific combination. Hence, this report underscores the critical need for comprehensive anatomical awareness among clinicians, particularly head and neck surgeons, to navigate these variations safely, improving patient outcomes.

## Introduction

The Sternocleidomastoid muscle serves as a pivotal anatomical landmark in the neck, dividing the region into the anterior and posterior cervical triangles. Typically, this muscle originates from two distinct heads. A sternal head arising from the anterior surface of the manubrium sternum, and a clavicular head originating from the superior surface of the medial third of the clavicle. The muscle subsequently inserts onto the mastoid process of the temporal bone [1-3]. Beyond its crucial role in anatomical orientation, the Sternocleidomastoid facilitates head rotation, lateral flexion, and assists in deep inspiration by elevating the sternum. The anatomical anomalies of this muscle can result in both functional impairment and distortion of the normal cervical anatomy [1].

The omohyoid muscle is characterized by its attachments to the hyoid bone and the scapula [4,5]. It consists of superior and inferior bellies connected by an intermediate tendon, which is typically covered by the Sternocleidomastoid muscle. The superior belly is situated within the anterior cervical triangle, while its inferior belly lies within the posterior cervical triangle, thereby contributing to the subdivision of these areas [4].

This report documents observed anatomical variations, specifically an unusual origin of the Sternocleidomastoid muscle and a flat morphology of the omohyoid muscle. Given their role as landmarks delineating cervical triangles, variations in these muscles necessitate clinician awareness for accurate diagnosis and safe interventions.

## Case presentation

During a routine anatomical dissection of the neck region in a formalin-fixed, 80-year-old male cadaver at the Anatomy Department of a tertiary care medical college, there were no notable scars in the neck region, and the cause of death was unremarkable for this case. The right and left neck regions were carefully dissected and examined for muscular variations, with findings documented via photography and detailed notes. The following anatomical variations were observed on the right side of the neck.

**Figure 1:**
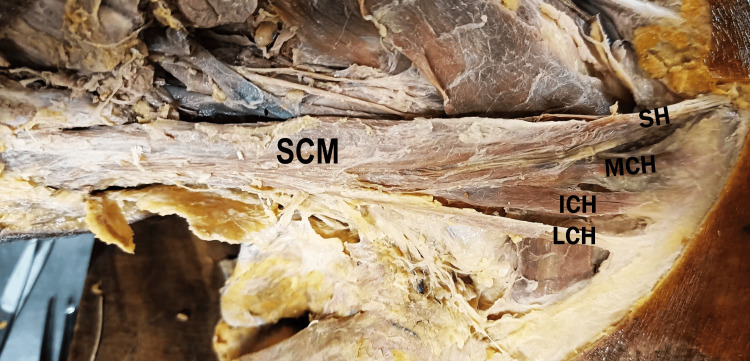
Right neck dissection showing the Sternocleidomastoid muscle with one sternal head (SH) and three clavicular heads: medial, intermediate, and lateral accessory heads (MCH, ICH, and LCH). SCM, Sternocleidomastoid; SH, sternal head of Sternocleidomastoid; MCH, medial clavicular head of Sternocleidomastoid; ICH, intermediate clavicular head of Sternocleidomastoid; LCH, lateral clavicular head of Sternocleidomastoid

The right Sternocleidomastoid muscle was found to originate from four distinct heads. A single sternal head arose from the anterior surface of the manubrium sternum, while three separate clavicular heads originated from the medial two-thirds of the clavicle. These heads ascended and fused into a single muscle belly that inserted onto the mastoid process of the temporal bone (Figure 1). The neurovascular supply to the muscle appeared consistent with typical anatomy.

**Figure 2:**
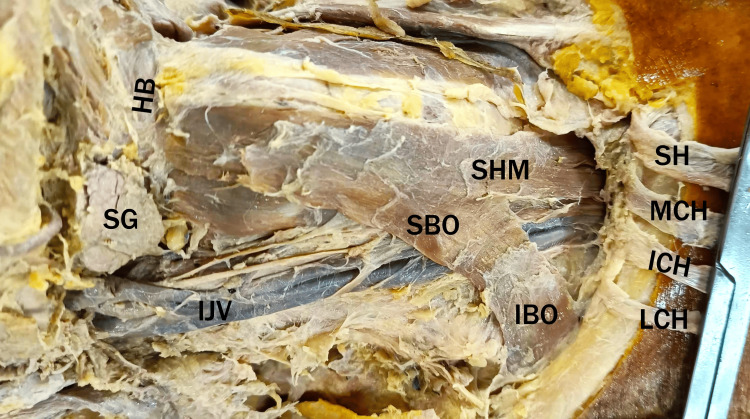
The right neck dissection showing the right omohyoid muscle. The Sternocleidomastoid muscle was cut and reflected to expose the omohyoid muscle fully. Note the superior belly of the omohyoid muscle (SBO) merging with the sternohyoid muscle (SHM)  at the lower aspect of the neck. SBO, superior belly of omohyoid; IBO, inferior belly of omohyoid; SG, submandibular gland; HB, hyoid bone; IJV, internal jugular vein; SH, sternal head of Sternocleidomastoid; MCH, medial clavicular head of Sternocleidomastoid; ICH, intermediate clavicular head of Sternocleidomastoid; LCH, lateral clavicular head of Sternocleidomastoid

In contrast to its usual structure, the right omohyoid muscle presented as thin, flat fibers. The superior belly merged indistinguishably with the sternohyoid muscle from the lower part of the neck to their common hyoid bone attachment. A notable finding was the absence of an intermediate tendon, resulting in a lack of clear demarcation between the superior and inferior bellies. Both bellies appeared thin and musculomembranous, particularly in the region covered by the Sternocleidomastoid muscle (Figure 2). The neurovascular supply to the omohyoid muscle appeared consistent with typical anatomy from the ansa cervicalis.

Inspection of the left side of the neck revealed that both the Sternocleidomastoid and omohyoid muscles exhibited typical anatomical morphology. This study was conducted per ethical standards, with cadaveric specimens sourced from the institution’s anatomy department under approved protocols for educational and research purposes.

## Discussion

The Sternocleidomastoid muscle is reported to have multiple attachments [3]. The variations in the attachment can be unilateral or bilateral, it may be a sternoclavicular attachment variation or a mastoid attachment [1]. The Sternocleidomastoid muscle is a key landmark dividing the anterior and posterior triangles of the neck and providing protection to vital neurovascular structures, including the internal jugular vein and brachial plexus [1,2]. Congenital torticollis, abnormal head posture, the use of Sternocleidomastoid muscle flaps in reconstructive surgery, and prevention of Frey's syndrome (the Sternocleidomastoid flap aims to prevent the misdirection of regenerating parasympathetic nerve fibers to the sweat glands) are well-established clinical applications of the Sternocleidomastoid [2]. Aberrations in its morphology, such as accessory heads as in our case, can alter anatomical relationships, making surgical procedures in the neck region, including neck dissections, central venous catheterization, and reconstructive surgeries, more challenging and increasing the risk of iatrogenic injury.

The Sternocleidomastoid and trapezius muscles develop from a shared embryonic muscle mass derived from branchial arch mesoderm and occipital myotomes. In case of atypical splitting or differentiation of this mesodermal mass can lead to the formation of supernumerary heads, most commonly affecting the clavicular origin [6]. The presence of four heads, as observed in this case, represents a significant departure from the typical two-headed morphology and underscores a complex developmental aberration.

Studies report multiple omohyoid variations [4,5]. Zhao et al. reported the absence of the omohyoid muscle [7]. These variations have been classified into double superior and inferior bellies (type I), Cleido-hyoideus (type II), short omohyoid with superior belly merging with sternohyoid (type III), and superior belly having additional fibers from the sternum (type IV) [4]. The reported variation of a flat-fibered right omohyoid lacking an intermediate tendon and exhibiting a superior belly merging with the sternohyoid aligns partly with previously classified type III (short omohyoid with superior belly merging with sternohyoid) but is distinct due to its flat, musculomembranous fibers and complete absence of an intermediate tendon, suggesting a novel subtype.

The omohyoid muscles are taking part in the anterior and posterior triangles of the neck and their subdivisions [4,5]. It is clinically vital, since it is a useful surgical landmark for the level III and level IV lymph nodes, identification of the internal jugular vein, neck reconstruction, and its association with cerebral hemodynamics, as it may compress the internal jugular vein in case of accessory heads [4]. Due to its importance in neck dissection and its adjacency to large vessels and the brachial plexus, an in-depth knowledge of the omohyoid muscle's variations is vital to head and neck surgeons.

## Conclusions

This report presents a novel combination of a right sternocleidomastoid muscle with four heads and a right omohyoid muscle characterized by flat fibers, absence of an intermediate tendon, and a superior belly merging with the sternohyoid. These variations can alter anatomical relationships, posing challenges for head and neck surgery. Preoperative imaging and detailed anatomical training can help surgeons anticipate such anomalies, enhancing patient safety. These findings encourage further studies on neck muscle variations to improve surgical outcomes.

## References

[1] Silawal S, Schulze-Tanzil G (2023). The sternocleidomastoid muscle variations: a mini literature review. Folia Morphol (Warsz).

[2] Sakthivel S, Dhakshnamoorthy N, G SN (2024). Accessory head of sternocleidomastoid muscle in Indian cadavers: a report of three cases. Cureus.

[3] Nayak SB, Soumya KV (2020). Sternocleidomastoid muscle can have multiple attachments. Surg Radiol Anat.

[4] Rai R, Ranade A, Nayak S, Vadgaonkar R, Mangala P, Krishnamurthy A (2008). A study of anatomical variability of the omohyoid muscle and its clinical relevance. Clinics (Sao Paulo).

[5] Hatipoğlu ES, Kervancioğlu P, Tuncer MC (2006). An unusual variation of the omohyoid muscle and review of literature. Ann Anat.

[6] Matsuoka T, Ahlberg PE, Kessaris N (2005). Neural crest origins of the neck and shoulder. Nature.

[7] Zhao W, Liu J, Xu J, Wang H (2015). Duplicated posterior belly of digastric muscle and absence of omohyoid muscle: a case report and review of literature. Surg Radiol Anat.

